# Quantitative and Qualitative Urinary Cellular Patterns Correlate with Progression of Murine Glomerulonephritis

**DOI:** 10.1371/journal.pone.0016472

**Published:** 2011-01-31

**Authors:** Junpei Kimura, Osamu Ichii, Saori Otsuka, Tomonori Kanazawa, Yuka Namiki, Yoshiharu Hashimoto, Yasuhiro Kon

**Affiliations:** 1 Laboratory of Anatomy, Biomedical Sciences, Graduate School of Veterinary Medicine, Hokkaido University, Sapporo, Japan; 2 Office for Faculty Development and Teaching Enriched Veterinary Medicine, Graduate School of Veterinary Medicine, Hokkaido University, Sapporo, Japan; University of Georgia, United States of America

## Abstract

The kidney is a nonregenerative organ composed of numerous functional nephrons and collecting ducts (CDs). Glomerular and tubulointerstitial damages decrease the number of functional nephrons and cause anatomical and physiological alterations resulting in renal dysfunction. It has recently been reported that nephron constituent cells are dropped into the urine in several pathological conditions associated with renal functional deterioration. We investigated the quantitative and qualitative urinary cellular patterns in a murine glomerulonephritis model and elucidated the correlation between cellular patterns and renal pathology.

Urinary cytology and renal histopathology were analyzed in BXSB/MpJ (BXSB; glomerulonephritis model) and C57BL/6 (B6; control) mice. Urinary cytology revealed that the number of urinary cells in BXSB mice changed according to the histometric score of glomerulonephritis and urinary albumin; however, no correlation was detected for the levels of blood urea nitrogen and creatinine. The expression of specific markers for podocytes, distal tubules (DTs), and CDs was detected in BXSB urine. Cells immunopositive for Wilms tumor 1 (podocyte marker) and interleukin-1 family, member 6 (damaged DT and CD marker) in the kidney significantly decreased and increased in BXSB versus B6, respectively. In the PCR array analysis of inflammatory cytokines and chemokines, *Il10*, *Cxcl2*, *C3*, and *Il1rn* showed relatively higher expression in BXSB kidneys than in B6 kidneys. In particular, the highest expression of *C3* mRNA was detected in the urine from BXSB mice. Furthermore, C3 protein and mRNA were localized in the epithelia of damaged nephrons.

These findings suggest that epithelial cells of the glomerulus, DT, and CD are dropped into the urine, and that these patterns are associated with renal pathology progression. We conclude that evaluation of urinary cellular patterns plays a key role in the early, noninvasive diagnosis of renal disease.

## Introduction

Lack of renal disease control is an inevitable problem in clinical medicine because the kidney is a nonregenerative organ. The global population of patients with end-stage renal disease (ESRD) has recently been increasing [1]. Several studies have indicated that chronic kidney disease (CKD) is strongly associated with ESRD progression [2]–[4], and the rapid increase in the number of patients with CKD has become a worldwide public health problem.

Chronic glomerulonephritis (CGN), which begins with glomerular lesions (GLs), is one of the major CKDs that is primarily caused by certain infections, drugs, and systemic disorders [2], [5]. In the early stages of CGN, glomerular immune-complex depositions cause GLs, such as capillary barrier disruption, which lead to ultrafiltration of plasma proteins or protein-associated factors [5]. Chronic GLs are thought to be converted into tubulointerstitial lesions (TILs) by ultrafiltration of several proteins and inflammatory cytokines or local hypoxia [5]. Eventually, CGN progresses to ESRD through a final common pathway in which progressive interstitial fibrosis is associated with tubular atrophy and peritubular capillary loss [5].

Recent studies have attempted to discover new biomarkers for the development of a new diagnostic strategy for CKD control, in which tissue injury markers such as inflammatory cytokines, chemokines, or slit diaphragm molecules are noted [6], [7]. The most suitable strategy for CKD control is the establishment of a noninvasive diagnostic method that can detect pathological conditions at the early stages; however, no protocol currently satisfies this requirement.

It has recently been suggested that loss of nephron constituent cells results in deterioration of renal function. The pathological correlations between podocyte loss and GLs are suggested in human and animal models [8]–[13]. Hara *et al.* detected podocytes and their fragments in the urine of patients with several glomerular diseases [14]–[18]. Moreover, Sato *et al.* demonstrated that podocyte mRNAs were detected in the urine of rats administered with drugs [19]. On the other hand, Ichii *et al.* demonstrated a correlation between distal tubular epithelial damage and TILs in murine CGN models, showing luminal epithelial deciduation (LED; the term “deciduation” means the dropping of epithelia into lumen) [20]. These reports suggest that damaged renal parenchymal cells are dropped into the urine as renal disease progresses. However, no study has reported on the quantitative and qualitative details of urinary cells derived from spontaneous animal models.

As the model for CGN, MRL/MpJ-*lpr*/*lpr*, NZB/WF1, and BXSB/MpJ-*Yaa* are widely used and these strains develop systemic autoimmune diseases such as increase of serum autoantibodies and vasculitis as well as glomerulonephritis. Especially, BXSB mice carry the mutant gene located on the Y chromosome, designated as *Yaa* (Y-linked autoimmune acceleration), and male mice show more severe glomerulonephritis than females. Therefore, this male CGN model could eliminate the effect of estrous cycle to autoimmune disease [21]. Andrews *et al.* demonstrated the deposits of immune complexes such as IgG and C3 in glomeruli from BXSB kidneys [22], indicating that BXSB mice can be used as a representative model of lupus nephritis. Furthermore, BXSB mice develop both GLs and subsequent TILs similar to human CGN pathology, and this strain was evaluated as the most appropriate model for the present study.

In this study, we analyzed the correlation between urinary cytology and CGN pathology. Our results indicate that renal parenchymal cells, including epithelia of the glomerulus, distal tubules (DTs), and collecting ducts (CDs), fall into the urine as CGN progresses. On the basis of these findings, we propose that evaluation of urinary cellular patterns should lead to the development of an early, noninvasive diagnostic method.

## Materials and Methods

### Ethical Statement

This study was carried out as part of a research project entitled “Analysis of the MRL/MpJ mice phenotypes.” This project includes the analysis of disease models such as autoimmune disease, CKD, and urogenital organ disease to develop new diagnosis methods. This research was approved by the Institutional Animal Care and Use Committee, which is convened at the Graduate School of Veterinary Medicine, Hokkaido University (approval no. 09-0129). The investigators adhered to *The Guide for the Care and Use of Laboratory Animals of Hokkaido University*, *Graduate School of Veterinary Medicine* (approved by the Association for Assessment and Accreditation of Laboratory Animal Care International).

### Animals and Sample Preparations

Male BXSB/MpJ-*Yaa* (BXSB) mice (*n* = 12) and C57BL/6 (B6) mice (*n* = 5) ages 3–6 months were purchased from Japan SLC, Inc. (Hamamatsu, Japan), and were maintained under specific pathogen-free conditions. The mice were subjected to deep anesthesia (pentobarbital sodium 60 mg/kg administered intraperitoneally), and urine was collected by bladder puncture to avoid contamination by lower urinary tract cells. Bladder urine was collected, and the animals were euthanized by exsanguination from the carotid arteries; subsequently, humoral and organ samples were collected.

### Serological and Urinary Analysis

For renal function evaluation, serum blood urea nitrogen (BUN) and creatinine (Cre) levels in all animals were determined using BUN-test-Wako and Creatinine-test-Wako (Wako Pure Chemical Industries, Osaka, Japan) according to the manufacturer's instructions. Urinary albumin was detected by SDS-polyacrylamide gel electrophoresis. Briefly, 3 µL of urine and 1 µg of bovine serum albumin were heated at 65°C for 5 min in 2× SDS sample buffer [100 mM Tris-HCl (pH 6.8), 20% glycerol, 4% SDS, 0.02% bromophenol blue, 12% 2-mercaptoethanol] and loaded on 12% polyacrylamide gel (e-PAGEL; ATTO Corporation, Tokyo, Japan). Electrophoresis was performed at 150 V in Tris-glycine buffer [25 mM Tris (pH 8.3), 192 mM glycine] containing 0.1% SDS for 2 h. Gels were stained with Quick CBB PLUS (Wako Pure Chemical Industries).

### Cytology of Urinary Cells

Two staining methods were performed to observe and identify urinary cell morphology. First, 100 µL of urine was immediately centrifuged at 1500 rpm for 5 min. Ninety microliters of supernatant urine was then removed, and 200 µL of 4% paraformaldehyde (PFA) was added. Urinary cells fixed by 4% PFA were centrifuged at 1500 rpm for 5 min, and 190 µL of supernatant was removed. The remaining 20 µL of urine sediments was placed on a glass slide, dried, and stained with hematoxylin-eosin (HE). Second, 100 µL of freshly obtained urine was centrifuged at 1500 rpm for 5 min, and 90 µL of supernatant was removed. The remaining 10 µL of urine sediments was stained with Sternheimer-Malbin (SM) stain. After staining, the urine sediments were placed on a glass slide and a coverslip was gently applied. The number of cells per field was counted and averaged in at least 5 fields of the HE-stained samples, and urinary cells were characterized using the 2 staining techniques mentioned above.

### Reverse Transcription and Polymerase Chain Reaction

For mRNA expression examination, total RNA from urine was purified using the SV Total RNA Isolation System (Promega, Madison, WI, USA). DNase-treated total RNAs were synthesized to cDNAs by a reverse transcription (RT) reaction by using the ReverTra Ace reverse transcriptase enzyme (Toyobo, Osaka, Japan) and oligo dT primers (Invitrogen, Carlsbad, CA, USA). Each cDNA, adjusted to 1.0 µg/mL, was used for the polymerase chain reaction (PCR) reaction with Ex Taq (Takara Bio, Tokyo, Japan) and the appropriate primer pairs including *Wt1*, *Nephrin*, *Podocin*, *Podocalyxin*, *Wt1*, *Serpinb7*, *Vwf*, *Aqp1*, *Slc12a1*, *Aqp2*, *Il10*, *Cxcl2*, *C3*, and *Il1rn*, as shown in Table 1. Nested PCR reactions were performed using 1/20 volume of the first PCR products with the primer pairs designed at the inside of the sequence between the first primer pairs. The amplified samples were electrophoresed with 1% agarose gel containing ethidium bromide and finally photographed under an ultraviolet lamp.

**Table 1:** Summary of specific gene primers.

Genes	Primer sequence (5′-3′)	Product size	Primer sequence (5′-3′)	Product size	Application	Specific expresion cell
(accession)	F: forward, R: reverse	(bp)	F: forward, R: reverse	(bp)		
Wt1	F: GCATGACCTGGAATCAGATG	383	F: GGTATGAGAGTGAGAACCACACG	137	Urine RT-PCR	Podocyte
(NM_144783)	R: TCTCTCGCAGTCCTTGAAGTC		R: AGATGCTGACCGGACAAGAG			
Nphs1	F: GATCCAGGTCTCCATCACTACC	432	F: AGGAGGATCGAATCAGGAATG	161	Urine RT-PCR	Podocyte
(NM_019459)	R: AAGGCCATGTCCTCATCTTC		R: GCGATATGACACCTCTTCCAG			
Nphs2	F: GACCAGAGGAAGGCATCAAG	496	F: AAGGTTGATCTCCGTCTCCAG	105	Urine RT-PCR	Podocyte
(NM_130456)	R: GTCACTGCATCTAAGGCAACC		R: TTCCATGCGGTAGTAGCAGAC			
Actn4	F: TCCAGGACATCTCTGTGGAAG	340	F: CCTTCAATGCACTCATCCAC	147	Urine RT-PCR	Podocyte
(NM_021895)	R: AAGGCATGGTAGAAGCTGGAC		R: TGTCCTCAGCATCCAACATC			
Vwf	F: ACAAGTGTCTGGCTGAAGGAG	316	F: TGCTGTGACACATGTGAGGAG	160	Urine RT-PCR	Endothelium
(NM_011708)	R: CACTGCATGGCGTTGATG		R: GCACATCCTCGATGTCAATG			
Serpinb7	F: GGCCTTCACCAAGACTGATAC	390	F: ACCAATGCAGGTTCTTGAGC	129	Urine RT-PCR	Mesangial cell
(NM_027548)	R: CCAGAGGCAATTCCAGAGAG		R: CTCCTATTGGTCCAGTCCATC			
Aqp1	F: GCATTGAGATCATTGGCACTC	351	F: GCTGGCGATTGACTACACTG	199	Urine RT-PCR	PT epithelium
(NM_007472)	R: CATCCAGGTCATACTCCTCCAC		R: ACTGGTCCACACCTTCATGC			
Slc12a1	F: CCACAAAGATTTGACCACTGC	325	F: CAGAACTGGAAGCAGTCAAGG	179	Urine RT-PCR	DT epithelium
(NM_183354)	R: CACCAAGGCACAACATTTCTC		R: AGGAGGAAGGTTCTTGGTCAG			
Aqp2	F: CCATGTCTCCTTCCTTCGAG	310	F: CGCCATCCTCCATGAGATTAC	110	Urine RT-PCR	CD epithelium
(NM_009699)	R: GGAGCAGCCGGTGAAATAG		R: TCAGGAAGAGCTCCACAGTC			
Cd3e	F: CCATCTCAGGAACCAGTGTAGAG	417	F: TGCCTCAGAAGCATGATAAGC	244	Urine RT-PCR	T cell
(NM_007648)	R: CATAGTCTGGGTTGGGAACAG		R: TTGGCCTTCCTATTCTTGCTC			
Ptprc	F: GAGGTGTCTGATGGTGCAAG	336	F: TGGAGGCTGAATACCAGAGAC	153	Urine RT-PCR	B cell
(NM_011210)	R: TCATCTGATTCAGGCTCACTCTC		R: TGCTCATCTCCAGTTCATGC			
Cd68	F: TGGATTCAAACAGGACCTACATC	388	F: CTACATGGCGGTGGAATACA	263	Urine RT-PCR	Macrophage
(NM_009853)	R: CTGGTAGGTTGATTGTCGTCTG		R: CAATGATGAGAGGCAGCAAG			
IL10	F: TGCTATGCTGCCTGCTCTTAC	186	-	-	Urine RT-PCR	-
(NM_010548)	R: CGGTTAGCAGTATGTTGTCCAG					
Cxcl2	F: TCAAGAACATCCAGAGCTTGAG	170	-	-	Urine RT-PCR	-
(NM_009140)	R: TCCAGGTCAGTTAGCCTTGC					
C3	F: TGCAGACTGAACAGAGAGCAG	134	-	-	Urine RT-PCR	-
(NM_009778)	R: CTCACAACACTTCCGAAGACC					
C3	F: CACTGGACCCAGAGAAGCTC	866	-	-	*In situ* hybiridization	-
(NM_009778)	R: GGATGTGGCCTCTACGTTGT					
Il1rn	F: TTGTGCCAAGTCTGGAGATG	174	-	-	Urine RT-PCR	-
(NM_031176)	R: TCTAGTGTTGTGCAGAGGAACC					

Primer sequences given on the left column are for the first PCR, and those on the right column are for the second PCR. PT: proximal tubule, DT: distal tubule, CD: collecting duct).

### Histological Analysis

The kidney samples for histology were fixed by 4% PFA at 4°C overnight. Paraffin sections (2 µm thick) were then prepared and stained with periodic acid Schiff (PAS). To assess the severity of glomerulonephritis, semiquantitative glomerular damage scoring was performed as previously described [23]. Briefly, 100 glomeruli per kidney was examined by using PAS-stained sections and scored from 0 to +4 according to the following criteria: 0, no recognizable lesion in glomeruli; +1, a little PAS-positive deposition, mild cell proliferation, mild membranous hypertrophy, and/or partial podocyte adhesion to the parietal layer of the renal corpuscle; +2, segmental or global PAS-positive deposition, cell proliferation, membranous hypertrophy, and/or glomerular hypertrophy; +3, the same as grade 2 with PAS-positive deposition in 50% of regions of glomeruli and/or severe podocyte adhesion to the parietal layer of the renal corpuscle; +4, disappearance of capillary and capsular lumina, global deposition of PAS-positive material, and/or periglomerular infiltration of inflammatory cells and fibrosis, based on the degrees of PAS-positive deposition, cell proliferation, membranous hypertrophy, podocyte adhesion to the parietal layer, disappearance of capillary and capsular lumina, and periglomerular infiltration of inflammatory cells and fibrosis. If, for example, 50 of 100 glomeruli were +1, 25 of 100 glomeruli were +2, 20 of 100 glomeruli were +3, and 5 of 100 glomeruli were +4, the semiquantitative score would be {(1×50/100) + (2×25/100) + (3×5/100) + (4×5/100)} ×100  = 180.

### Immunohistochemical and Immunofluorescence Analyses

Immunostaining for Wilms tumor 1 (WT1) and interleukin-1 family, member 6 (IL-1F6) was performed according to the following procedure. The paraffin sections were deparaffinized and incubated in citrate buffer (pH 6.0) for 20 min at 105°C for antigen retrieval. After cooling, slides were soaked in methanol containing 3% H_2_O_2_ for 15 min at room temperature to remove internal peroxidase. After being washed, sections were blocked by 10% normal goat serum (for WT1) or 10% normal donkey serum (for IL-1F6) for 60 min at room temperature and incubated with rabbit polyclonal IgG antibodies for WT1 (1∶1000; Calbiochem/EMD, Darmstradt, Germany) or goat polyclonal antibodies for IL-1F6 (1∶400; R&D Systems, Minneapolis, MN, USA) overnight at 4°C. After washing 3 times in phosphate-buffered saline (PBS), sections were incubated with biotin-conjugated goat anti-rabbit IgG antibodies for WT1 (SABPO® kit, Nichirei, Tokyo, Japan) or donkey anti-goat IgG antibodies for IL-1F6 (Santa Cruz Biotechnology, Santa Cruz, CA, USA) for 30 min at room temperature, washed, and incubated with streptavidin-biotin complex (SABPO® kit) for 30 min. The sections were then incubated with 3,3′-diaminobenzidine tetrahydrochloride-H_2_O_2_ solution. Finally, the sections were slightly counterstained with hematoxylin. Immunostaining for C3 was performed according to the following procedure. In brief, deparaffinized 2-µm-thick paraffin sections were incubated in 0.1% pepsin/0.2 M HCl for 5 min at 37°C for antigen retrieval. After being washed, sections were pretreated with 0.25% casein/0.01 M PBS for 60 min at room temperature and incubated with goat polyclonal IgG antiserum for C3 (1∶800; MP Biomedicals, Solon, OH, USA) overnight at 4°C. After washing 3 times with PBS, the sections were incubated with TRITC-labeled rabbit anti-goat IgG antibodies (1∶200; Zymed/Invitrogen) for 30 min at room temperature and washed again. For nuclear staining, sections were incubated with Hoechst 33342 (1∶200; Wako Pure Chemical Industries) for 30 min. Finally, the sections were examined under a confocal laser scanning microscope (LSM700; Zeiss, Thornwood, NY, USA).

### *In Situ* Hybridization Analysis

cRNA probes for C3 were synthesized in the presence of digoxigenin-labeled UTP by using a DIG RNA Labeling Kit in accordance with the manufacturer's protocol (Roche Diagnostics, Mannheim, Germany). The primer pairs for making each probe are shown in Table 1. Deparaffinized, proteinase K–digested sections were incubated with a prehybridization solution and then with hybridization buffer containing 50% formamide, 10 mM Tris-HCl (pH 7.6), 200 mg/mL RNA, 1× Denhardt's solution (0.02% bovine serum albumin, 0.02% polyvinylpyrrolidone, and 0.02% Ficoll PM400; Amersham Pharmacia, Uppsala, Sweden), 10% dextran sulfate, 600 mM NaCl, 0.25% SDS, 1 mM EDTA (pH 8.0), and sense or antisense RNA probe (final concentration, 0.2 µg/mL) for 24 h at 58°C. The sections were then incubated with 0.2% polyclonal sheep anti-digoxigenin Fab fragments conjugated to alkaline phosphatase (1∶400; Nucleic Acid Detection Kit, Roche Diagnostics) for 24 h at room temperature. The signal was detected by incubating the sections with a color substrate solution (Roche Diagnostics) containing nitroblue tetrazolium/X-phosphate in a solution composed of 100 mM Tris-HCl (pH 9.5), 100 mM NaCl, and 50 mM MgCl_2_ in a dark room overnight at room temperature.

### PCR Array Analysis

To identify the factors that exacerbate the disease, PCR array analysis was performed and the relative expression of 84 inflammatory cytokines, chemokines, and their receptors were examined. Total RNAs were purified from the kidneys of 3-month-old male C57BL/6 and BXSB/MpJ mice, which were stored in RNAlater solution (Ambion/Applied Biosystems, Foster City, CA, USA), using TRIzol reagent (Invitrogen). After purification of the total RNAs with an RNeasy Micro Kit (Qiagen, Germantown, USA), the RNAs were treated with Turbo DNase (Ambion) for DNA digestion and then repurified. Five micrograms of total RNA was synthesized to cDNA by using the RT^2^ PCR Array First Strand Kit (SuperArray, Frederick, MD, USA). PCR array analysis was performed using 10 µL of cDNA solution, Mouse Inflammatory Response and Autoimmunity PCR RT^2^ Profiler™ PCR Array (SuperArray), and a MX 3000 thermal cycler (Stratagene, La Jolla, CA, USA).

### Statistical Analysis

Results were expressed as the mean ± standard error and statistically analyzed using a nonparametric Mann–Whitney *U* test (*P*<0.05). The correlation between 2 parameters was analyzed using Spearman's correlation test (*P*<0.05).

## Results

### Cytological Observation of Urinary Cells

**Figure 1:**
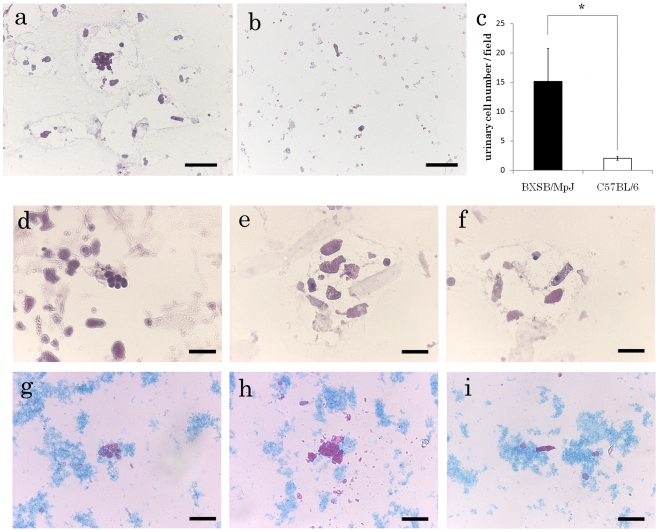
Cytology and the number of urinary cells in BXSB/MpJ and C57BL/B6. (a and b) Comparison of urinary smears from BXSB/MpJ (a) and C57BL/6 (b) mice. Bar  = 50 µm. Urinary cell numbers in BXSB/MpJ mice are higher than those in C57BL/6 mice. (c) Numbers of urinary cells in BXSB/MpJ and C57BL/6 mice. *, significantly different from C57BL/6 mice (Mann–Whitney *U* test, *P*<0.05); *n* = 11. (d–i) Morphology of urinary cells in BXSB/MpJ mice stained with HE (d–f) and SM (g–i). Small round cells (d and g), homogeneous and amorphous cell components (e and h), and columnar cells (f and i) are observed. Bar  = 50 µm.

To assess the number and morphology of urinary cells, urine sediment smears were examined using HE and SM stains. The urinary cell numbers in BXSB mice were significantly higher than those in B6 mice (Fig. 1a–c). In the urine from BXSB mice, several kinds of urinary cells were observed: small round cells (Fig. 1d and g), homogeneous and amorphous cell components (Fig. 1e and h), and columnar cells with basophilic cytoplasm (Fig. 1f and i). Among these cell types, small round cells showed an aggregation pattern, and this was also observed in control mice.

### Correlations between Urinary Cell Number and Renal Pathology

**Figure 2:**
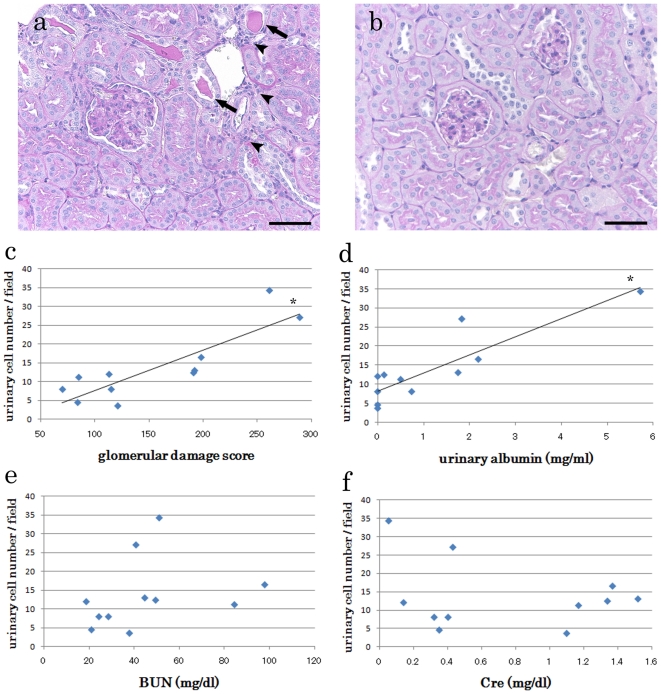
Comparison between renal condition and urinary cell number. (a and b) Representative PAS-stained kidney sections from BXSB/MpJ (a) and C57BL/6 (b) mice. Expansion of mesangial matrix, proliferation of mesangial cells, dilation of tubules by urinary cast (arrow), and perivascular cell infiltration (arrowhead) are observed in the BXSB kidney. Bars  = 50 µm. (c) Relationship between glomerular damage score and urinary cell number. *P*<0.05, *r* = 0.802 (Spearman's correlation test); *n* = 11. (d) Relationship between urinary albumin and urinary cell number. *P*<0.05, *r* = 0.837 (Spearman's correlation test); *n* = 11. (e) Relationship between BUN level and urinary cell number; *n* = 11. (f) Relationship between Cre level and urinary cell number; *n* = 11. BUN and serum Cre levels do not correlate with urinary cell number.

BXSB mice showed GLs and TILs, namely, the expansion of mesangial matrix, proliferation of mesangial cells, dilated tubules by urinary casts, and perivascular cell infiltration (Fig. 2a and b). Glomerular damage score was used as an index of GLs and was comparable to urinary cell number, suggesting that the number of urinary cells significantly increased with glomerular damage score (Fig. 2c). In addition, urinary cell number significantly correlated with urinary albumin; however, no correlation was detected with BUN and Cre (Fig. 2d–f).

### Identification of Urinary Cell Types

For urinary cell identification, urinary mRNA detection was performed. Markers of renal parenchymal cells, including podocytes (*Wt1*, *Nphs1*, *Nphs2*, *Actn4*); mesangial cells (*Serpinb7*); vascular endothelium (*Vwf*); proximal tubular epithelial cells (*Aqp1*); distal tubular epithelial cells (*Slc12a1*); and CD epithelial cells (*Aqp2*), including T cells (*CD3e*), B cells (*Ptprc*), and macrophages (*CD68*), were used. Table 2 shows that the expression of *Wt1*, *Nphs1*, *Actn4*, *Slc12a1*, and *Aqp2* was detected in the urine from BXSB mice at a high rate. In addition to markers of renal epithelium, *Vwf* was detected in a few urine samples from BXSB mice. Although perivascular infiltration of inflammatory cells was observed in BXSB kidneys (Fig. 2a), inflammatory cell markers were not detected in BXSB urine.

**Table 2:** Expression of various nephron constituent cell markers in the urine from 12 BXSB/MpJ mice.

	Case 1	Case 2	Case 3	Case 4	Case 5	Case 6	Case 7	Case 8	Case 9	Case 10	Case 11	Case 12
*Wt1*	+	-	-	-	-	-	-	-	-	-	+	+
*Nphs1*	+	-	-	+	-	-	+	-	++	-	+	-
*Nphs2*	-	-	-	-	-	-	-	-	-	-	-	-
*Actn4*	+	-	-	-	-	-	-	-	+	-	-	+
*Vwf*	-	-	-	-	-	+	-	+	-	+	-	-
*Serpinb7*	-	-	-	-	-	-	-	-	-	-	-	-
*Aqp1*	-	-	-	-	-	-	-	-	-	-	-	-
*Slc12a1*	+	+	+	+	+	+	+	+	+	+	+	+
*Aqp2*	+	+	+	+	+	+	+	+	+	+	+	++
*Cd3e*	-	-	-	-	-	-	-	-	-	-	-	-
*Cd68*	-	-	-	-	-	-	-	-	-	-	-	-
*Ptprc*	-	-	-	-	-	-	-	-	-	-	-	-

++, detected at first PCR;

+, detected at second PCR; -, not detected.

### Histological Evidence of Renal Parenchymal Cell Loss

**Figure 3:**
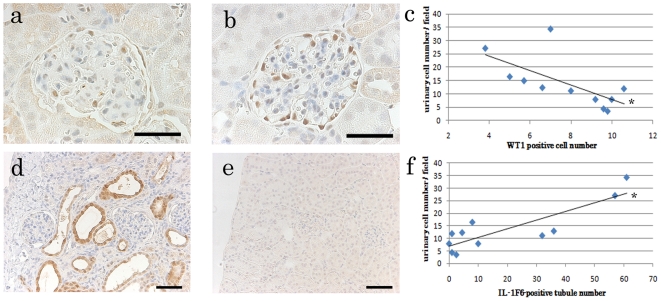
Localization of WT1 and IL-1F6 proteins and urinary cell number. (a and b) Immunohistochemistry of BXSB/MpJ (a) and C57BL/6 (b) kidneys. WT1-positive reactions are observed in podocyte nuclei. The number of WT1-positive cells in the BXSB/MpJ kidney is higher than that in the C57BL/6 kidney. Bars  = 50 µm. (c) Relationship between the number of WT1-positive cells and urinary cell number. *P*<0.05, *r* = −0.662 (Spearman's correlation test); *n* = 11. (d and e) Immunohistochemistry for IL-1F6 in BXSB/MpJ (d) and C57BL/6 (e) kidneys. IL-1F6-positive reactions are observed in damaged tubules. Bars  = 50 µm. (f) Relationship between the number of IL-1F6-positive tubules and urinary cell number. *P*<0.05, *r* = 0.712 (Spearman's correlation test); *n* = 11.

To confirm the urinary deciduation of podocytes, DT epithelium, and CD epithelium, immunohistochemical analysis of WT1 (podocyte marker) and IL-1F6 (marker of damaged DT and CD) was performed. WT1 was localized in podocyte nuclei in the glomerulus, and the number of glomerular WT1-positive cells in BXSB kidneys was significantly lower than that in B6 kidneys (Fig. 3a and b). Additionally, there was a significant inverse correlation between urinary cell number and the number of glomerular WT1-positive cells in BXSB kidneys (Fig. 3c). IL-1F6 was localized in epithelial cells from DTs and CDs showing tubular dilations or epithelial deciduation (Fig. 3d and e). The number of urinary cells was significantly correlated with the number of IL-1F6–positive tubules (Fig. 3f).

### Selection of Inflammatory Markers for Detection of Urinary Cells

For the development of inflammatory urine cell markers derived from the kidney, a PCR array targeting 84 inflammatory cytokines and chemokines and their receptors was analyzed using kidneys from male B6 and BXSB mice. Genes of the chemokine (C-X-C motif) ligand *(Cxcl)* and chemokine (C-C motif) ligand *(Ccl)* and their receptors *(Ccr and Cxcr)* were upregulated in BXSB mice. We found that interleukin-10 *(Il10)*, chemokine (C-X-C motif) ligand 2 *(Cxcl2)*, complement component 3 *(C3)*, and interleukin-1 receptor antagonist *(Il1rn)* showed particularly high expression levels in BXSB mice (Table 3). Among 4 highly upregulated mRNAs (*Il10*, *Cxcl2*, *C3*, and *Il1rn*), the expression of *C3* was detected in the urine from BXSB/MpJ at a high rate (Table 4).

**Table 3:** Summary of the results of PCR array analysis targeting aggravating factors of chronic glomerulonephritis.

Ranking	Symbol	Accession no.	BXSB/B6
expressing higher level			
1	*Il10*	NM_010548	8.75
2	*Cxcl2*	NM_009140	8.46
3	*C3*	NM_009778	5.58
4	*Il1rn*	NM_031176	4.53
5	*Cxcl1*	NM_008176	4.03
6	*C3ar1*	NM_009779	3.41
7	*Il8rb*	NM_009909	3.36
8	*Ccl2*	NM_011333	3.36
9	*Ccl17*	NM_011332	3.20
10	*Ccl7*	NM_013654	3.14
11	*Ccr3*	NM_009914	3.10
12	*Il23r*	NM_144548	3.07
13	*Tlr2*	NM_011905	2.87
14	*C4b*	NM_009780	2.83
15	*Ccl3*	NM_011337	2.83
16	*Tnf*	NM_013693	2.81
17	*Il1b*	NM_008361	2.77
18	*Itgb2*	NM_008404	2.58
19	*Ccl11*	NM_011330	2.51
20	*Ccl8*	NM_021443	2.51
21	*Il23a*	NM_031252	2.51
22	*Ccl4*	NM_013652	2.41
23	*Ccr2*	NM_009915	2.33
24	*Ccr1*	NM_009912	2.33
25	*Fos*	NM_010234	2.23
26	*Il6*	NM_031168	2.11
27	*Ltb*	NM_008518	1.99
28	*Tlr7*	NM_133211	1.79
29	*Cxcl5*	NM_009141	1.78
30	*Ccl20*	NM_016960	1.77

Values are fold increase compared to B6. BXSB, BXSB/MpJ; B6, C57BL/6.

**Table 4:** Summary of results showing the expression of *Il10*, *Il1rn*, *C3*, and *Cxcl2* mRNAs in the urine from BXSB/MpJ mice.

	*Il10*	*Il1rn*	*C3*	*Cxcl2*
Case 1	-	-	+	-
Case 2	-	-	-	-
Case 3	-	-	-	-
Case 4	-	-	+	-
Case 5	-	-	-	-
Case 6	-	-	+	-
Case 7	+	-	+	-
Case 8	-	-	-	-
Case 9	-	+	+	-
Case 10	-	-	-	-
Case 11	-	-	-	-
Case 12	-	-	-	-

+, positive;

-, negative.

### Localization of C3-Producing Cells in the Kidney

**Figure 4:**
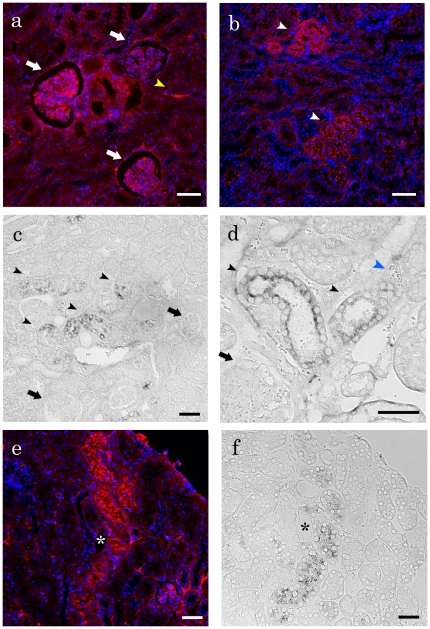
C3 protein and *C3* mRNA expression in the urine and kidneys from BXSB/MpJ mice. (a and b) Immunofluorescence for C3. Positive C3 reactions are observed in the glomerular capillary rete (a, white arrow), tubular epithelial cells (b, white arrowhead), and vascular endothelia (a, yellow arrowhead). Additionally, several C3-positive tubules tend to be localized in the same cortical regions (b). Bars  = 50 µm. (c and d) *In situ* hybridization for *C3* mRNA. Positive reactions are observed in the cytoplasm of tubular epithelial cells (c, black arrowhead). Similar to C3 protein staining, several *C3* mRNA-positive tubules are localized in the same cortical regions. On the other hand, the glomerulus (black arrow) and vascular endothelium (blue arrowhead) are not stained (c and d). Bars  = 50 µm. (e and f) Immunohistochemistry and *in situ* hybridization for C3 protein and *C3* mRNA in serial sections. C3 protein (e) and *C3* mRNA (f) show colocalization in the same tubles. *, the same vessel. Bars  = 50 µm.

Immunofluorescence analysis of the kidney showed that complement C3 protein was localized in the glomerulus, tubular epithelial cells, and vascular endothelium (Fig. 4a and b). To examine whether C3 protein was synthesized or deposited, *in situ* hybridization of *C3* mRNA was also performed. Figure 4c and d shows that positive reactions were detected in the epithelia of cortical renal tubules. Several positive tubules tended to localize in the same cortical regions. Furthermore, the colocalization of *C3* mRNA and its protein was confirmed by the serial sections (Fig. 4e and f).

## Discussion

### Relationship between Urinary Cell Number and CGN Pathology

It has been clinically recognized that cells derived from the kidney, such as cellular casts, appear in the urine of patients with renal disease, and these cell components indicate the pathological conditions of the kidney. In the present study, we elucidated a significant positive correlation between urinary cell number and indices of renal pathology, such as glomerular damage score and urinary albumin levels, by using spontaneous animal models.

In humans, a prominent histological feature of CGN is cellular hyperplasia in the glomerulus as well as glomerular inflammatory diseases in experimental conditions caused by both proliferation of mesangial cells and infiltration of leukocytes [24]. However, the present study suggests that urinary deciduation was derived from podocytes, but not mesangial cells or inflammatory cells. Recent studies have indicated that infiltrated inflammatory cells produce various reactive oxygen species, pro-inflammatory cytokines, matrix metalloproteinases (MMPs), and transforming growth factors, which modulate local response and increase inflammation [25]. In particular, factors such as transforming growth factor-β and MMPs were reported to play an important role in the loss of cell adhesion molecules through the E-cadherin and integrin families [26], [27]. These findings suggest that cellular hyperplasia in the glomerulus and subsequent inflammatory reactions might contribute to the detachment of podocytes but not of other cells.

Common pathological characteristics between human and animal models in the case of glomerular diseases, such as CGN and diabetic nephropathy, include urinary leakage of proteins, such as albumin (albuminuria), caused by disruption of the blood-urine barrier [28], [29]. In the present study, urinary cell number correlated with urinary albumin but not with BUN and Cre levels, indicating that urinary albumin correlated with urine cell number rather than serological values showing renal function. Although measurement of BUN and Cre levels is the most widely used method for renal diagnosis in the clinical field, neither BUN nor Cre can be used as a precise indicator of renal function because of a lack of sensitivity and specificity [30]. The present and previous studies suggest that evaluation of urinary cell number is a sensitive and specific method for diagnosing renal pathology, especially blood-urine barrier disruption. Furthermore, cell number in the urine also seemed to be a more specific marker than urinary albumin because the latter could be elevated by contamination of the urine with secretions from various glands in the lower urinary tract, such as sex accessory glands.

### Urinary Cell Types

Identification of urinary cells could lead to a detailed understanding of renal pathological conditions. From microscopic observation, the urinary cell numbers in BXSB mice were significantly higher than those in B6 mice. However, excessive deciduation of bladder epithelial cells increases the number of urinary cells. Bladder epithelial cells are characterized as transitional epithelium. Therefore, these cells dropping into the urine might show an aggregation pattern. We consider the small round cells to be bladder epithelial cells because this cell type exhibited an aggregation pattern and was also observed in control mice. In addition, these cells were observed less frequently than the other cell types. From these findings, we also inferred that the homogeneous and amorphous cell components or the columnar cells with basophilic cytoplasm might be derived from the kidney. To identify the details of these cell types, we performed urine PCR analysis for cell-specific genes.

In Urine PCR analysis, *Wt1*, *Nephrin*, *Actn4*, *Slc12a1*, and *Aqp2* mRNA were detected in BXSB urine at a high rate. Furthermore, these mRNAs tended to be detected in the urine from mice with more severe renal conditions (Table 5). On the other hand, the expression of *Vwf* was also detected in a few BXSB urine samples. Although endothelial cells from glomerular capillaries might drop into the urine because of glomerular epithelium (podocyte) damage, the expression of podocyte marker was not detected in *Vwf*-positive urine. In addition, the indices of renal damage from their kidney showed low levels. Therefore, we considered the bladder urine to be contaminated by the endothelial cells as a result of vessel damage by bladder puncture. From These findings, it was strongly suggested that podocytes and DT and CD epithelia fall into urine as the renal pathological condition of glomerulonephritis progresses.

**Table 5:** Summary of results detecting pathological parameters and urinary cell patterns in the urine from 12 BXSB/MpJ mice and 5 C57BL/6 mice.

	Urinary cell number	Glomerular damage score	Urinary albumin (mg/ml)	BUN (mg/dl)	Cre (mg/dl)	WT1 positive cell number	IL-1F6 positive tubule number	mRNA expression			
								Podocyte marker	DT/CD marker	Other marker	Inflammatory cytokine
BXSB case 1	34.3	261	5.72	51.1	0.05	7.0	61.0	*Nephrin, Wt1, Actn4*	*Aqp2, Slc12a1*	-	*C3*
BXSB case 2	8.0	70	0.74	28.6	0.40	9.2	0.0	-	*Aqp2, Slc12a1*	-	-
BXSB case 3	11.2	85	0.50	84.4	1.17	8.0	32.0	-	*Aqp2, Slc12a1*	-	-
BXSB case 4	16.5	198	2.19	97.8	1.37	5.0	8.0	*Nephs1*	*Aqp2, Slc12a1*	-	*C3*
BXSB case 5	4.5	84	0.00	21.1	0.35	9.6	1.0	-	*Aqp2, Slc12a1*	-	-
BXSB case 6	8.0	115	0.00	24.4	0.32	10.0	10.0	-	*Aqp2, Slc12a1*	*Vwf*	*C3*
BXSB case 7	27.1	289	1.83	40.8	0.43	3.8	57.0	*Nephs1*	*Aqp2, Slc12a1*	*Serpinb7*	*C3, Il10*
BXSB case 8	12.0	113	0.00	18.8	0.14	10.6	1.0	-	*Aqp2, Slc12a1*	*Vwf*	-
BXSB case 9	15.0	192	1.75	44.7	1.52	6.2	36.0	*Nephs1, Actn4*	*Aqp2, Slc12a1*	-	*C3, Il1rn*
BXSB case 10	3.6	121	0.00	37.9	1.10	9.8	2.5	-	*Aqp2, Slc12a1*	*Vwf*	-
BXSB case 11	12.4	191	0.14	49.5	1.34	5.7	4.5	*Nephs1, Wt1*	*Aqp2*	-	-
BXSB case 12	no data	261	5.71	129.8	2.31	3.8	22.0	*Actn4, Wt1*	*Aqp2, Slc12a1*	-	-
Average	13.9	156	1.55	52.4	0.86	7.4	19.6	/	/	/	/
B6 case1	1.3	3.0	0.12	44.1	0.37	19.0	0.0	*Wt1*	*Aqp2*	-	-
B6 case2	2.5	0.0	0.00	30.7	0.09	15.2	0.0	-	-	-	-
B6 case3	2.5	5.0	0.00	39.6	0.58	14.8	0.0	-	-	-	-
B6 case4	2.5	6.0	0.00	52.1	0.75	12.6	0.0	-	*Aqp2, Slc12a1*	-	-
B6 case5	1.6	3.0	0.00	32.8	0.55	13.0	0.0	-	-	-	-
Average	2.1	3.4	0.024	39.9	0.47	14.9	0.0	/	/	/	/

BXSB, BXSB/MpJ; B6, C57BL/6; DT, distal tubule; CD, collecting duct; -, negative; /, not applicable.

Podocytes, highly differentiated cells lining the outside of the glomerular capillaries, are composed of a body with extending primary processes that further branch into foot processes separated by a slit diaphragm. Recently, it has been suggested that the effacement of podocytes started from disruption of foot processes and/or the slit diaphragm is associated with the development of proteinuric renal diseases, and their mRNAs are detected in the urine of renal disease patients [31]–[33]. In the present study, molecular and morphological analyses showed that the loss of podocytes (podocytopenia) [34] or urinary excretion of podocytes [14]–[18] was associated with progression of renal pathology in glomerulonephritis. Interestingly, among various makers for podocytes, *Nphs1* was detected at a high rate. According to Nakatsue *et al.*, urinary Nphs1 protein, but not Nphs2 protein, was detected in the urine at the early stages of rabbit Heyman nephritis [35], indicating that urinary Nphs1 protein is a useful tool for early diagnosis in the case of Heyman nephritis, although other podocyte markers such as *Wt1*, *Nphs2*, and *Actn4* are suggested to be helpful for the diagnosis of various kidney diseases [31]–[33]. These data therefore suggest that injured podocytes have different patterns of protein expression in each kidney disease, indicating that evaluation of podocyte deciduation using appropriate podocyte markers in each renal disease is essential for accurate and early diagnosis.

Ichii *et al.* demonstrated the correlation between TILs and LED in the distal segment [20], indicating that epithelia from damaged DT and CD expressing IL-1F6 fall into the urine. In the present study, DT and CD markers, but not proximal tubule (PT) markers, were detected in the urine from BXSB mice. IL-1F6 is known as a member of the IL-1 gene family, and its product has been identified as a member of the IL-1 cytokine family, which regulates inflammation by mediating the expression of various cytokines, chemokines, nitric oxide synthases, and MMPs [36]. In the kidney, IL-1F6 is associated not only with cellular infiltrations but also with changes in epithelial morphology [20]. In epithelial cells, the downregulation of epithelial markers and upregulation of mesenchymal markers are known as epithelial-to-mesenchymal transitions (EMTs), and the transitions of renal tubular epithelial cells have been shown to cause the progression of interstitial fibrosis [36]. Furthermore, it is suggested that PT epithelia that undergo EMT migrate to the tubulointerstitial space as transformed matrix-producing cells [37]. On the other hand, injured DT and CD epithelia are reported to fall into the tubular lumen but not into the tubulointerstitial space. From these findings, we inferred that the EMT mechanism might differ between the proximal and distal segments; namely, injured PT cells undergoing EMT eventually migrate to the tubulointerstitial space, whereas injured DT and CD cells move to LED. These findings suggest that evaluation of urinary DT and CD epithelia leads to the prognosis of TILs.

### Complement and Renal Pathology

For PCR array analysis, the upregulations of chemokines and their receptors were elucidated in BXSB kidneys. *Il10*, *Cxcl2*, *C3*, and *Il1rn* were highly upregulated in the BXSB kidney; interestingly, *C3* in particular was detected in BXSB urine. The complement system is the major effector of the humoral arm of the immune system. C3, which plays a pivotal role in the complement cascade, is the most abundant complement protein in the circulation. The majority of C3 is synthesized in the liver, but numerous other tissue sources of complement have been discussed. In the kidney, various cell types are capable of producing C3 *in vitro* and *in vivo* [38]–[40]. Furthermore, recent reports have indicated that C3 synthesized without liver may be a more important mediator of inflammation and immunological injury in the kidney than plasma C3 derived from the liver [41]. Our immunofluorescence and *in situ* hybridization analyses showed that damaged nephrons synthesized *C3* mRNA and protein. Furthermore, *C3* mRNA was detected in the urine from BXSB mice at a high rate. These results suggest that damaged cortical tubular epithelia synthesizing C3 fall into the urine.

The present study showed that some BXSB nephrons showing local C3 synthesis tended to become TILs. Once the complement cascade is activated by the presence of C3, generation of a sublytic concentration of C5b-9 alters renal epithelial cell function, inducing morphological changes, upregulation of collagen gene expression, and production of inflammatory cytokines [42], [43]. The data further suggest that C3a decreases the expression of E-cadherin protein and increases the expression of both α-smooth muscle actin protein and collagen type I mRNA in tubular epithelial cells [44]. These findings suggest that C3 plays an important role in the EMT. The EMT of tubular epithelial cells may lead to subsequent TILs, including LEDs and interstitial fibrosis.

In conclusion, we demonstrated that urinary cells reflect renal disease progression, such as podocyte effacement and DT/CD tubule damage, suggesting that a system for detecting urinary cells is a useful tool for the early, noninvasive diagnosis of several renal diseases. Additional studies for the development of a detection system for urinary cells are necessary to further animal and human health.
